# Early Detection of Ventilator-Associated Pneumonia in a Neurosurgical Patient: Do Biomarkers Help Us?

**DOI:** 10.7759/cureus.78567

**Published:** 2025-02-05

**Authors:** Tobias Bexten, Stefan Wiebe, Melindi Brink, Dominik Hinzmann, Golo-Sung Haarmeyer

**Affiliations:** 1 Clinic for Interdisciplinary Intensive Medicine and Intermediate Care, Helios Dr. Horst Schmidt Kliniken Wiesbaden, Wiesbaden, DEU; 2 Clinic for Anesthesiology and Intensive Care Medicine, Klinikum Rechts der Isar, Munich, DEU; 3 Clinic for Internal Intensive Care, Paracelsus Medical University, Nuremberg Hospital, Nuremberg, DEU

**Keywords:** interleukin (il)-6, neurosurgical intensive care unit, procalcitonin (pct), serum biomarkers, ventilator-associated pneumonia (vap)

## Abstract

Background: Ventilator-associated pneumonia (VAP) remains a significant complication in patients undergoing mechanical ventilation. It is particularly prevalent in neurosurgical patients, contributing to high morbidity and healthcare costs. Early diagnosis and timely treatment are crucial for preventing the progression of VAP. However, diagnosing VAP remains challenging because no diagnostic tool or biomarker can reliably confirm the condition. Nevertheless, biomarkers remain the most frequently used surrogates for VAP. Therefore, this study aimed to evaluate the predictive value of interleukin-6 (IL-6), procalcitonin (PCT), and C-reactive protein (CRP) and look at relevant clinical markers for the early detection of VAP. Additionally, the impact of the use of IL-6 or PCT/CRP on clinical decision-making in the diagnosis and management of VAP in neurosurgical patients was explored.

Methods: In this retrospective single-center study, we screened 1,817 neurosurgical intensive care patients between January 2020 and December 2023. In the first step, the ability of IL-6, PCT, and CRP to predict VAP was tested. We distinguished microbiologically confirmed VAP (mcVAP), suspected VAP (suspVAP ), and non-VAP. In the second step, two cohorts were compared: patients monitored with IL-6 alone and those monitored with PCT and CRP. Here, we compared the relevance of these markers for treatment decisions, antibiotic usage, and clinical parameters.

Results: A total of 240 neurosurgical patients fulfilled the eligibility criteria: 155 in the IL-6 group and 85 in the PCT/CRP group. The IL-6 level on the day of mcVAP and suspVAP was 106 pg/mL (IQR: 58.3-259.0) and 112 pg/mL (IQR: 58.3-259), respectively, whereas it was 33.55 ng/L (IQR: 19.6-59.2) in non-VAP patients (p<0.001; η² = 0.14, AUC 0.82 and 0.81, respectively). PCT also showed significant differences, although with a small effect size (η² = 0.008, p = 0.010). For CRP, no significant differences were observed (p = 0.317). In the IL-6 group, the start of treatment did not differ from that in the PCT/CRP group. The duration of antibiotic administration was slightly shorter in the IL-6 group than in the control group, although the differences were not statistically significant (6.25 (±3.73) and 5.9 (±2.23) days versus 7.37 (±4.95) and 7.67 (±2.65) days; p = 0.339 and p = 0.214, respectively).

Conclusion: While PCT has diagnostic value, IL-6 has superior predictive value for VAP, which is reflected in its high effect size and shorter duration of antibiotic treatment, although the difference was not significant. This study suggests that incorporating IL-6 as a routine biomarker may improve the early recognition of VAP, potentially optimizing treatment strategies in the neurosurgical ICU setting. However, the major limitation lies in the study’s retrospective design, which limits its generalizability.

## Introduction

Ventilator-associated pneumonia (VAP) is a frequent complication in patients who are exposed to invasive mechanical ventilation for at least 48 hours [1]. It is associated with a mortality of 12% [2,3], a prolonged time of invasive ventilation, and attributable health costs of 40,000 USD [4,5]. The incidence rate of 5-67% [6,7] represents a wide distribution depending on the diagnostic criteria and the patient’s baseline characteristics. The highest rates can be observed in burn-injured patients, followed by those with brain injuries [8].

Most commonly, VAP is defined as new, persistent, or progredient infiltrates in the chest X-ray, plus two of the following criteria: 1) white blood cells over 10/nL or less than 4/nL, 2) fever over 38°C, and/or 3) purulent secretion [9,10].

More recently, the US Centers for Disease Control (CDC) has proposed an updated definition to provide a more detailed definition. It includes general inflammatory and pulmonary signs, such as new-onset or altered-quality sputum, dyspnoea/tachypnoea/cough, rales, and altered gas exchange. Furthermore, the CDC divides "ventilator-associated events" (VAEs) into "ventilator-associated conditions" (VACs) and "infection-related ventilator-associated complications" (IVACs). This definition and the differentiations are not intended for clinical diagnosis but rather for disease surveillance [11].

However, diagnosing VAP remains challenging because no single diagnostic tool or biomarker can reliably confirm the condition. Consequently, surrogate biomarkers such as procalcitonin (PCT) and C-reactive protein (CRP) are commonly used to guide antibiotic initiation.

PCT is a 14.5-kDa peptide that serves as a precursor of calcitonin. Its serum levels rise in response to an infectious stimulus with a delay of four to six hours and halve daily under infection control by host immunity or antibiotic therapy [12,13]. For VAP, PCT values are elevated at the time of admission to the intensive care unit in patients who later develop VAP [14]. In the diagnosis of VAP, PCT has a sensitivity of 41-71% and a specificity of 73-100% [15].

CRP serum levels rise 10-12 hours after an infectious stimulus, reaching a maximum serum level 36-50 hours after the stimulus. Like PCT, CRP serum levels decrease significantly, with a half-life of 19 hours when the inflammatory stimulus is under control [12,16]. CRP indicates patients at risk for VAP with a cut-off value at 72 hours after intubation of 108 mg/L (AUC 0.912) [17] and has a sensitivity of 75.2% and specificity of 95.7% for the diagnosis of VAP [18].

In contrast, one of the most rapid parameters used to detect inflammation is interleukin-6 (IL-6) [12]. IL-6 is a cytokine released by macrophages, monocytes, endothelial cells, and mesenchymal cells to induce an acute phase reaction involving T and B cells and to cause the synthesis of acute-phase reactants in the liver, including CRP [19]. In response to an inflammatory stimulus, increased IL-6 serum levels were detected 24-48 hours before clinical evidence was obtained [12]. Its peak serum level is reached within six hours after the stimulus [15]. In a more recent multicenter trial, Zobel et al. demonstrated that, among a panel of serum markers, IL-6, PCT, and CRP were able to indicate patients at low risk for severe COVID-19 progression [20]. Another study by Ramirez et al. revealed that IL-6 is the only marker that can distinguish between microbiologically confirmed VAP and suspected VAP [21].

Still, it is not yet fully understood whether the choice of biomarkers affects the timing of VAP treatment and its impact on clinical outcomes. Therefore, this study aims to address three critical questions: (1) What is the predictive value of IL-6, CRP, and PCT for diagnosing VAP? (2) Is there a difference in biomarker levels when a microbiological pathogen is identified? (3) Does it matter if we use IL-6, PCT, or CRP as routine markers for early detection of VAP and antibiotic usage? To our knowledge, this is the first study to investigate the role of PCT, CRP, and IL-6 levels, particularly in relation to their effect on clinical outcomes.

## Materials and methods

We included neurosurgical patients admitted to the surgical ICU of the Helios Dr. Horst Schmidt Clinic in Wiesbaden, Germany, a tertiary referral hospital, either via the emergency department or postoperatively following major neurosurgical interventions. The observation period was from 01/01/2020 to 12/31/2023. The exclusion criteria were age less than 18 years, death within the first 48 hours of admission, incomplete baseline data documentation, disseminated infection, and immunosuppressant therapy, such as chemotherapy and interferon therapy. The study was designed as a retrospective single-center study at a tertiary referral hospital.

VAP was defined as ventilation for at least 48 hours, new, persistent, or progredient infiltrates in the chest X-ray, in combination with two out of three of the following criteria: 1) leucocytes > 10,000 or < 4,000/μL or 2) fever > 38°C or purulent secretion [9,10] and the presence of at least one positive microbiological culture obtained from tracheal secretions (microbiologically confirmed VAP (mcVAP)). In cases with a high mucus load, bronchoalveolar lavage was performed, contributing to microbiological confirmation. Suspected VAP was defined as meeting the criteria above without microbiological confirmation (suspVAP). On the day of study inclusion, we recorded sex, age, reason for admission, surgery performed, comorbidities, and the SAPS score. Daily assessment was performed, including clinical diagnostic criteria for VAP, antibiotic use, or the presence of other infections.

The overall observation period for VAP patients was 20 days after admission. The four days before mcVAP and suspVAP were defined as days -4, -3, -2, and -1, and the days after the diagnosis of VAP were defined as days 1, 2, 3, and 4. The day of VAP diagnosis was defined as day 0. During January 2020 and April 2022, IL-6 was the preferred daily marker; from then on, the PCT/CRP ratio was recorded daily. Microbiology findings, X-rays, temperatures, white blood cell (WBC) counts, P/F ratios, and albumin levels were also recorded if available.

Statistical analysis

Statistical significance was defined at an alpha level of <5%. Metric variables are indicated as the mean (±SD) and/or median (IQR). The differences in means or medians were tested with Student’s t-test, the Mann-Whitney U test, ANOVA, or the Kruskal‒Wallis test for metric variables as needed. The distribution of the data was tested via the Shapiro-Wilk test. Categorial variables were tested with the chi-square test. Cohen’s d (d), rank-biserial correlation (rₛ), eta squared (η²), or Cramer’s V (V) demonstrate the effect size as required [22]. Receiver operating characteristic (ROC) curves were used to illustrate the area under the curve (AUC), sensitivity, and specificity of each parameter in the prediction of VAP, including the odds ratio (OR) and the 95% confidence interval (CI). The optimum cut-off value was demonstrated as the threshold [23]. The statistical analysis was performed with JASP 0.18.2 and R 4.4.1 and Julius.ai (Caesar Labs, Inc., San Francisco, CA) for the graphs.

## Results

Population

**Figure 1:**
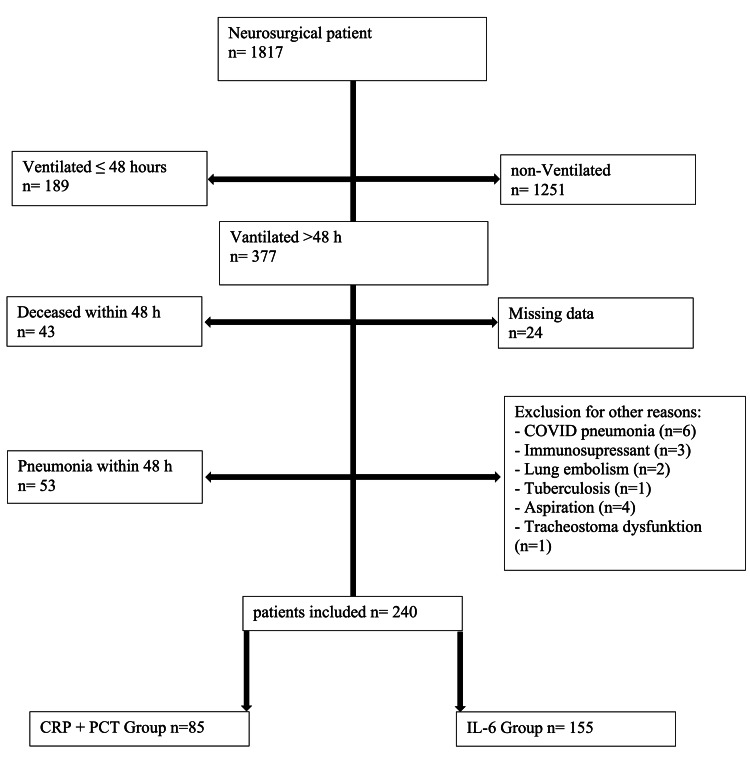
Exclusion tree The exclusion tree outlines the selection process of 1,817 neurosurgical patients. The final cohort of 240 patients was divided into the CRP + PCT group (n = 85) and the IL-6 group (n = 155).

A total of 1,817 neurosurgical patients in the ICU were screened. Of those, 1,440 were either not ventilated or were ventilated for less than 48 hours. Other reasons for exclusion were pneumonia within 48 hours after intubation (n=53), missing data (n=24), and other factors (n=17). After excluding those who did not fulfill the eligibility criteria, we included 240 patients for further investigation (Figure 1). We included 155 patients in the IL-6 group and 85 in the CRP/PCT group.

Intracerebral bleeding, affecting 196 patients (82%), was the primary reason for admission, followed by malignant infarction in 17 patients (7%) and 13 patients with traumatic injury (5%). Cardiovascular disease was the most common comorbidity, affecting 150 patients (63%), followed by substance abuse in 30 patients (12.6%) (Table 1).

**Table 1:** Baseline characteristics Baseline characteristics of patients with microbiologically confirmed ventilator-associated pneumonia (mcVAP), suspected ventilator-associated pneumonia (suspVAP), and patients without ventilator-associated pneumonia (non-VAP). Significant differences in baseline characteristics, indicated by P value (effect size), were observed in age (0.038* (0.029)), sex (0.001* (0.200)), ventilation hours (<0.001* (0.100)), and length of stay (0.001* (0.037)). In terms of diagnosis, differences were observed in malignant infarction (0.020* (0.181)), and in operative procedures, significant differences were found in decompressive craniotomy (0.044* (0.161)).

Baseline	mcVAP n=63	suspVAP n=30	non-VAP n=147	Total n=240	p-value (effect size)
Age (Years) (SD)	62.41 (±15.24)	70.67 (±11.38)	65.96 (±14.12)	65.62 (±14.45)	0.038* (0.029)
Sex F/M (%)	22/41 (35/65)	7/23 (23/77)	74/73 (50.5/49.5)	103/137 (43/57)	0.001* (0.200)
Ventilation (hours) (SD)	384.3 (±229.26)	326.47 (±164.32)	231.12 (±199.54)	283.52 (±214.11)	<0.001* (0.100)
Length of stay (hours)	580.2 (±342.85)	561.97 (±257.37)	435.77 (±362.29)	489.1 (±351.15)	0.001* (0.037)
SAPS at admission (SD)	41.33 (±12.28)	41.11 (±10.76)	39.23 (±12.78)	40.02 (±12.4)	0.127
Diagnosis (% of Total)					
Intracerebral bleeding	51 (21.3)	26 (10.8)	119 (49.6)	196 (81.7)	0.751
Traumatic	3 (1.3)	3 (1.3)	7 (2.9)	13 (5.4)	0.495
Localized infection	0 (0)	1 (0.4)	3 (1.3)	4 (1.7)	0.427
Tumor	0 (0)	0 (0)	5 (2.1)	5 (2.1)	0.199
Malignant infarction	9 (3.8)	0 (0)	8 (3.3)	17 (7.1)	0.020* (0.181)
Epileptic status	0 (0)	0 (0)	2 (0.8)	2 (0.8)	0.528
Hypoxic brain injury	0 (0)	0 (0)	3 (1.3)	3 (1.3)	0.383
Comorbidity (% of Total)					
Cardiovascular	38 (15.9)	21 (8.8)	91 (38.1)	150 (62.8)	0.655
Respiratorydisease	4 (1.7)	1 (0.4)	10 (4.2)	15 (6.3)	0.114
Kidney disease	3 (1.3)	1 (0.4)	4 (1.7)	8 (3.3)	0.752
Substance abuse	8 (3.3)	4 (1.7)	18 (7.5)	30 (12.6)	0.988
Others	8 (3.3)	5 (2.1)	14 (5.8)	27 (11.3)	0.484
Major operative procedures (% of Total)					
Decompressive craniotomy or craniectomy	27 (11.3)	12 (5)	39 (16.3)	78 (32.5)	0.044* (0.161)
Decompressive hemicraniectomy	14 (5.8)	1 (0.4)	25 (10.4)	40 (16.7)	0.07
Aneurysm clipping/coiling	3 (1.3)	1 (0.4)	4 (1.7)	8 (3.3)	0.752
External Ventricular Drain	17 (7.1)	14 (5.8)	67 (27.9)	98 (40.8)	0.168
Spinal decompression	2 (0.8)	2 (0.8)	3 (1.3)	7 (2.9)	0.386
Tumor resection	0 (0)	0 (0)	8 (3.3)	8 (3.3)	0.073

mcVAP/suspVAP versus non-VAP

A total of 63 patients (26%) met the criteria for mcVAP, 30 patients (12.5%) met the criteria for suspVAP, and 147 patients (61.25%) met the criteria for non-VAP. The time of mcVAP diagnosis was 5.56 (±3.87), and the time of suspVAP diagnosis was 6.47 (±4.33) days after intubation.

The ICU length of stay (LOS), duration of ventilation, and 30-day mortality rates are summarized in Table 1. Patients with mcVAP were significantly younger than those with suspVAP were (p = 0.014), whereas no significant differences in age were detected between the mcVAP group and the non-VAP group (p = 0.098 and p = 0.121, respectively). Compared with the non-VAP group, the mcVAP and suspVAP groups had significantly longer ICU stays and prolonged ventilation times (Table 1).

We found no further significant differences in admission diagnosis or comorbidities or SAPS scores between mcVAP or suspVAP patients and non-VAP patients, except for malignant infarction, for which the difference was between mcVAP patients and suspVAP patients (Tab. 1). At admission, there were no significant differences in IL-6, WBC, PCT, CRP, the P/F ratio, or body temperature (data not displayed).

Day of mcVAP and suspVAP (day 0)

**Figure 2:**
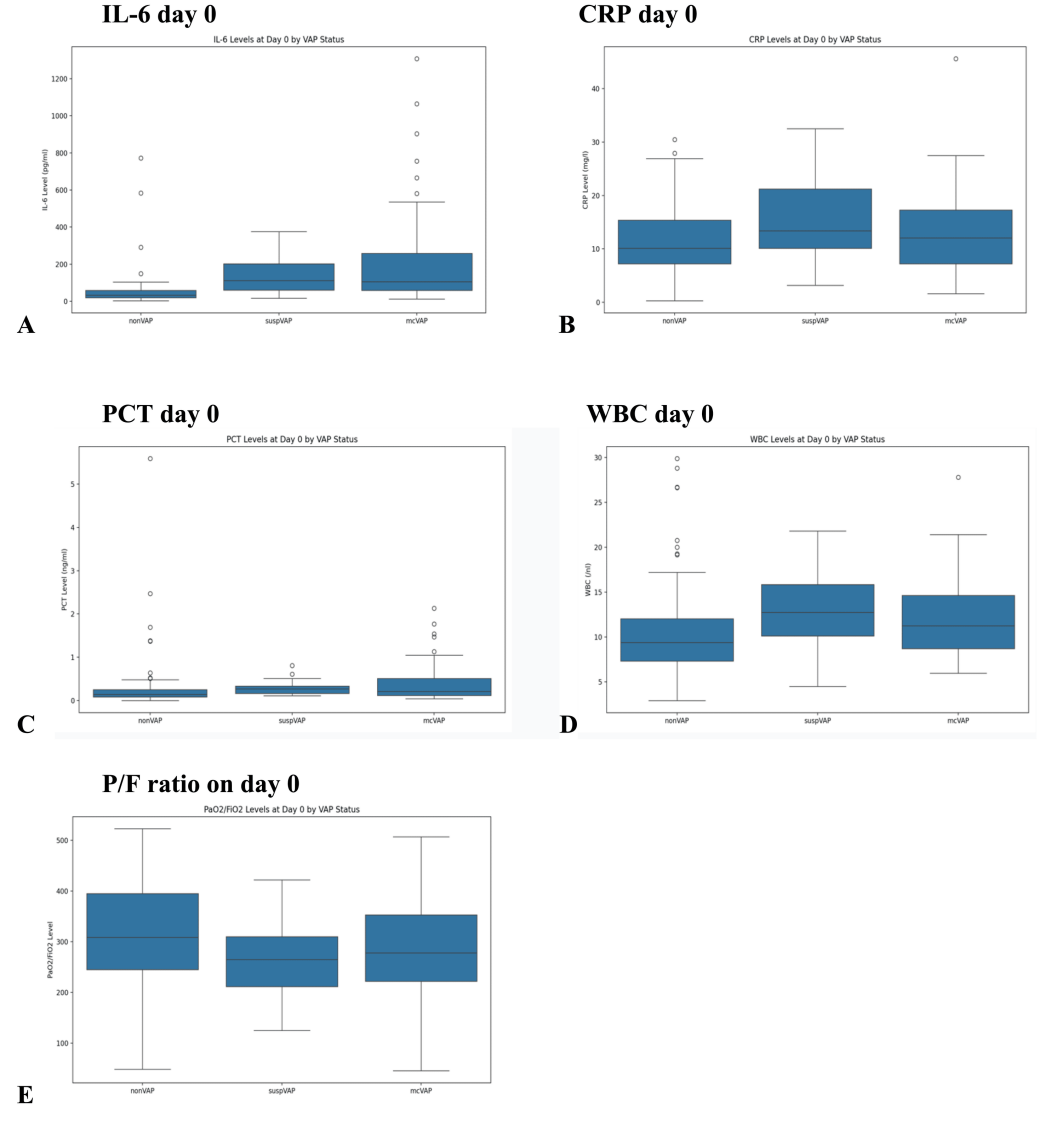
Interval plots on day 0 by the VAP status Interval plots of (A) IL-6 levels (pg/mL) on day 0 for non-VAP, suspVAP, and mcVAP; (B) CRP levels (mg/L) on day 0 across the three groups; (C) PCT levels (ng/mL) on day 0; (D) WBC levels (×10³/µL) on day 0; and (E) PaO_2_/FiO_2_ ratio on day 0 illustrating the differences in oxygenation status. IL-6: interleukin-6, PCT: procalcitonin, CRP: C-reactive protein, WBC: white blood cell, VAP: ventilator-associated pneumonia

On the day of mcVAP and suspVAP diagnoses, IL-6 levels were significantly higher in the VAP groups than in the non-VAP groups (η² = 0.140, p < 0.001). PCT also showed significant differences, although with a small effect size (η² = 0.008, p = 0.010). For CRP, no significant differences were observed (p = 0.317). For day 0, we also looked at the WBC and the P/F ratio, which differed significantly, with small effect sizes (p = 0.001, η² = 0.023, and p = 0.026, η² = 0.034, respectively) (Figure 2, Table 2). No significant differences were detected between the biomarker levels in mcVAP and suspVAP. The median, IQR, are shown in Table 2.

**Table 2:** Marker on day 0 of mcVAP or suspVAP Levels of various markers on day 0 across patients with suspected VAP (suspVAP), microbiologically confirmed VAP (mcVAP), and those without VAP (non-VAP). For significance testing, the Mann-Whitney test was performed, with the effect size indicated as a rank-biserial correlation. Significant differences were observed in IL-6, PCT, WBC, and PaO_2_/FiO_2_, but not in CRP, with the highest effect size seen in IL-6. IL-6: interleukin-6, PCT: procalcitonin, CRP: C-reactive protein, WBC: white blood cell, VAP: ventilator-associated pneumonia

Marker	Group	Median (IQR)	p-value (effect size)
IL6 pg/nL	suspVAP	112.0 (60.2-202.0)	<0.001 (0.64)*
	mcVAP	106.0 (58.3-259.0)	<0.001 (0.62)*
	non-VAP	33.5 (19.6-59.2)	Reference
PCT mg/L	suspVAP	0.3 (0.2-0.5)	0.007 (0.41)*
	mcVAP	0.2 (0.1-0.7)	0.044 (0.37)*
	non-VAP	0.1 (0.1-0.4)	Reference
CRP mg/dL	suspVAP	13.7 (10.1-21.2)	0.138
	mcVAP	12.2 (5.6-16.1)	0.397
	non-VAP	9.3 (5.6-12.7)	Reference
WBC/nL	suspVAP	13.4 (10.8-15.9)	<0.001 (0.42)*
	mcVAP	12.2 (9.1-15.2)	0.003 (0.27)*
	non-VAP	9.6 (8.0-12.1)	Reference
PaO_2_/FiO_2_	suspVAP	252.0 (195.0-310.0)	0.012 (-0.31)*
	mcVAP	278.0 (212.5-339.0)	0.118
	non-VAP	299.0 (211.0-395.5)	Reference

Biomarkers in repetitive assessment

**Figure 3:**
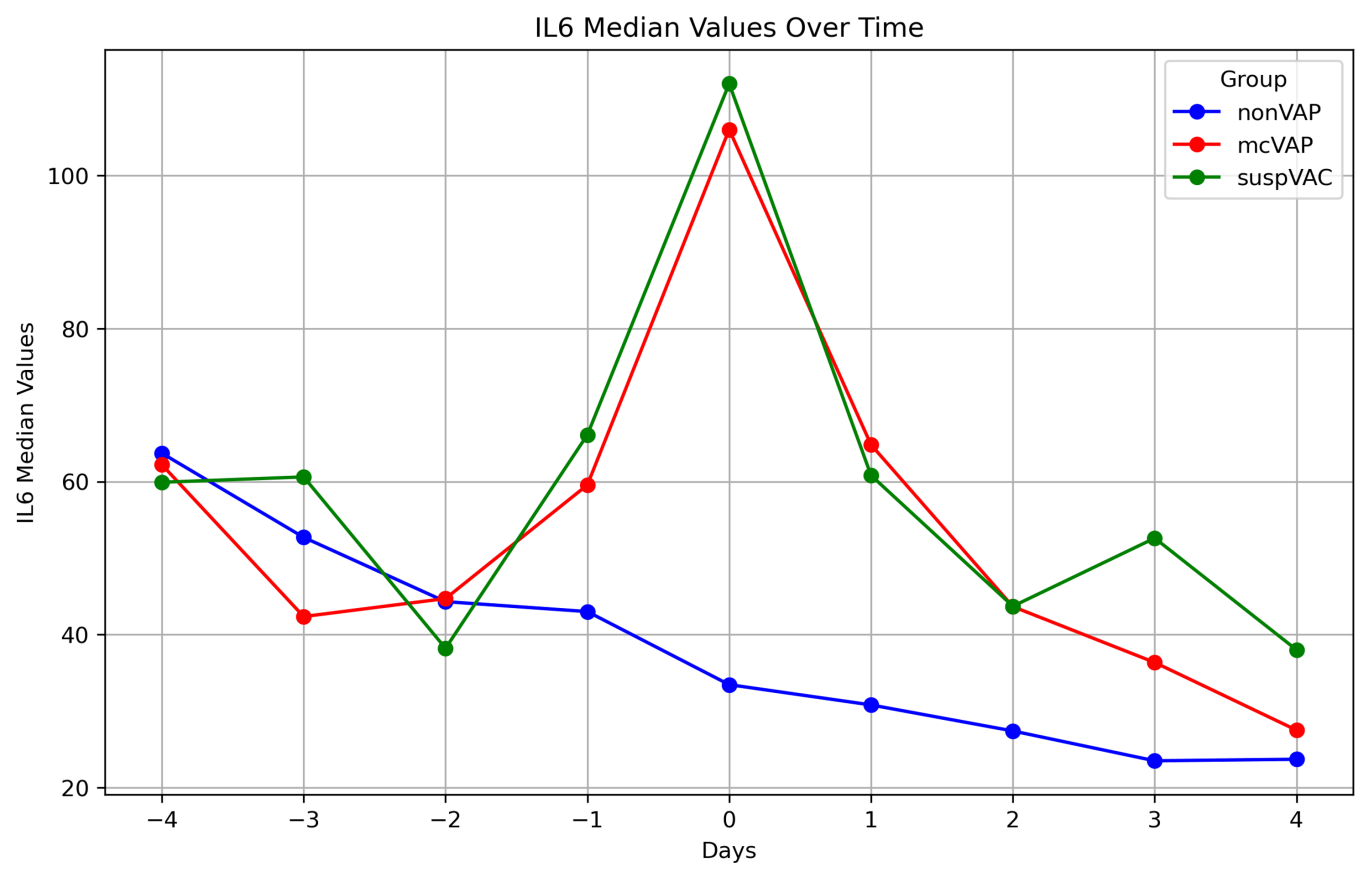
IL-6 in the repetitive assessment The graphs illustrate the median IL-6 levels over time (days -4 to +4) in three patient groups: non-VAP, mcVAP, and suspVAP. Median values are plotted for each day, highlighting trends and differences between groups. IL-6: interleukin-6, VAP: ventilator-associated pneumonia

**Figure 4:**
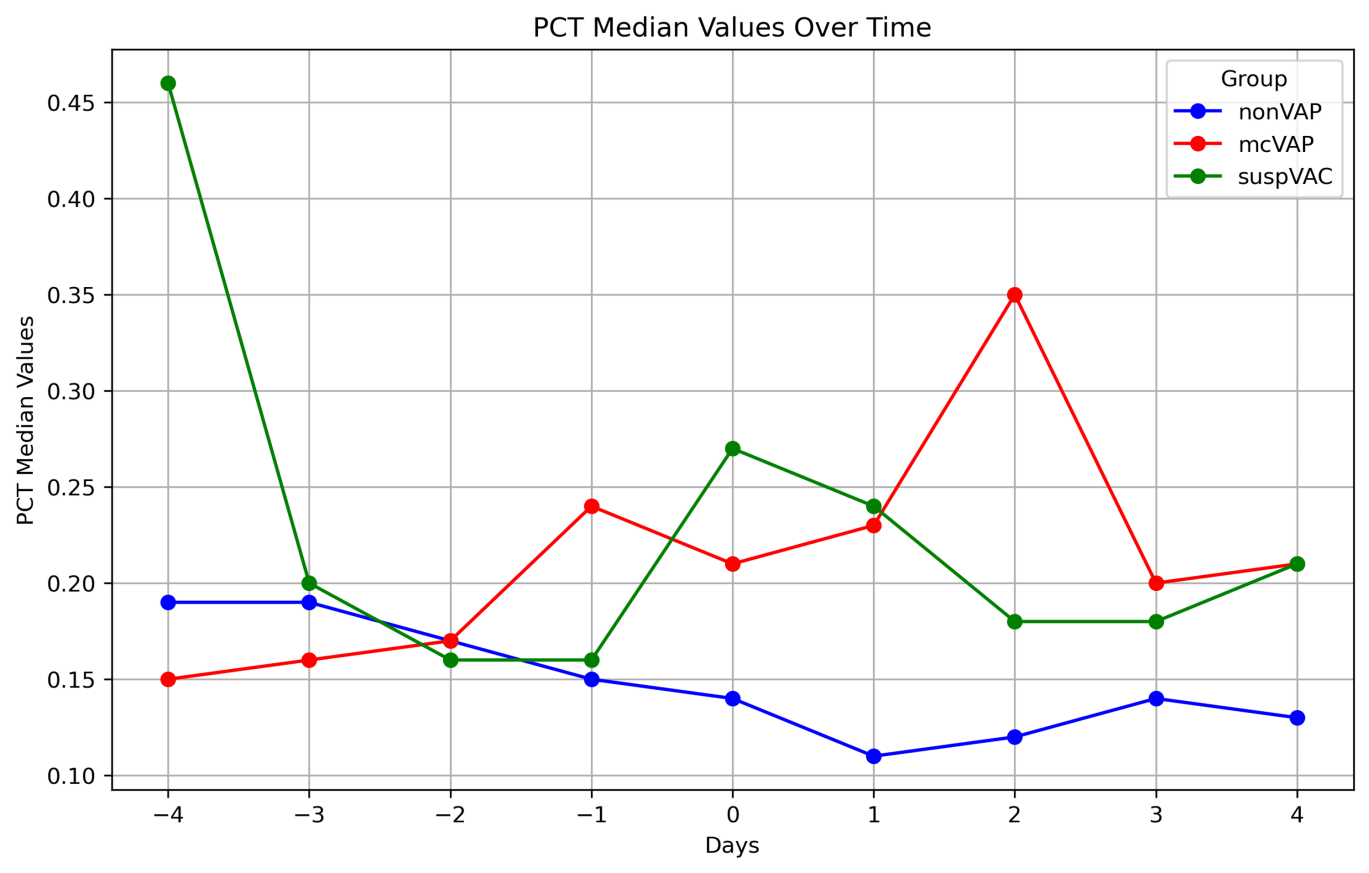
PCT in the repetitive assessment The graph illustrates the median PCT over time (days -4 to +4) in three patient groups: non-VAP, mcVAP, and suspVAP. Median values are plotted for each day, highlighting trends and differences between groups. PCT: procalcitonin, VAP: ventilator-associated pneumonia

**Figure 5:**
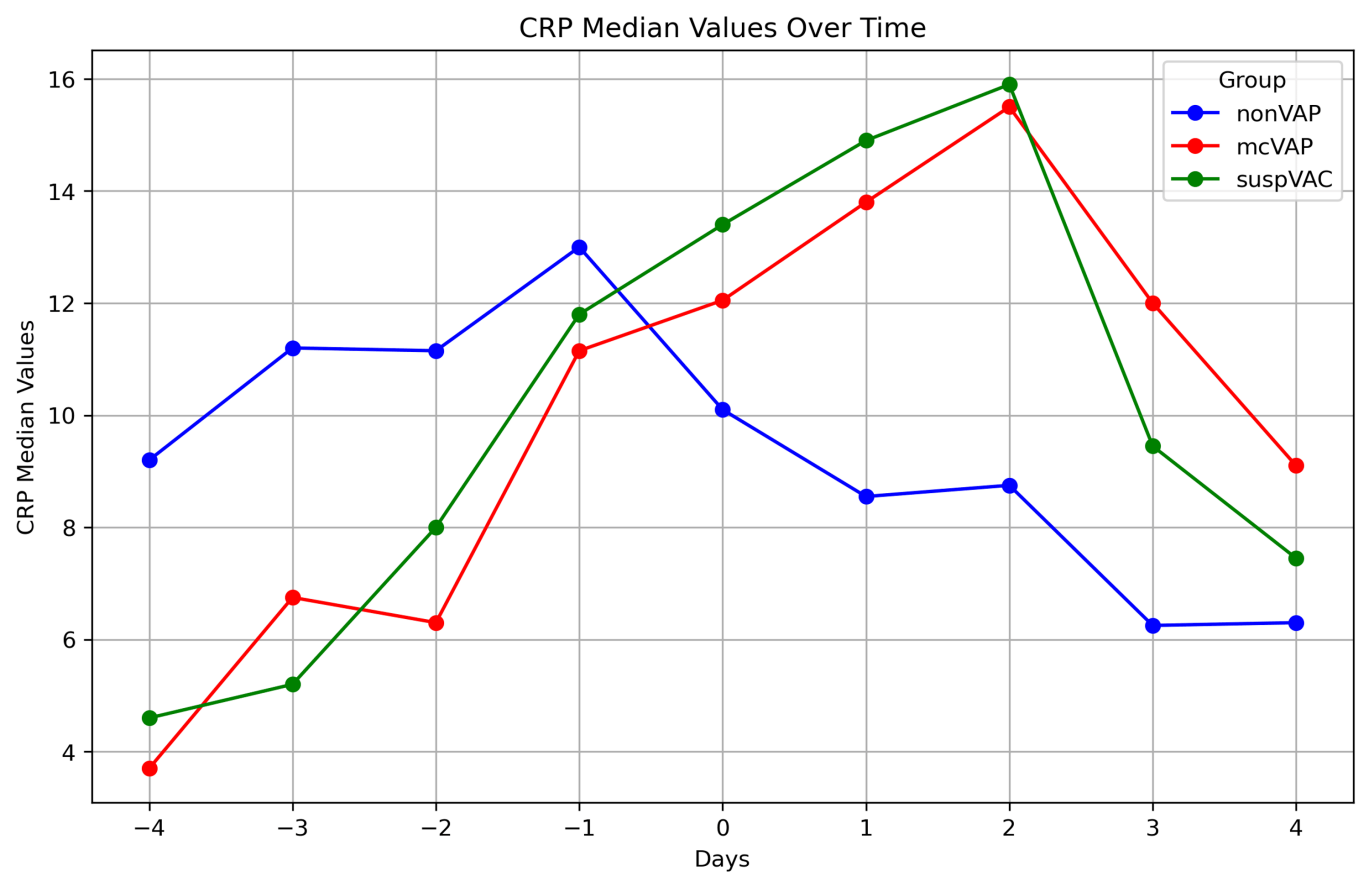
CRP in the repetitive assessment The graph illustrates the median the CRP level over time (days -4 to +4) in three patient groups: non-VAP, mcVAP, and suspVAP. Median values are plotted for each day, highlighting trends and differences between groups. CRP: C-reactive protein, VAP: ventilator-associated pneumonia

**Figure 6:**
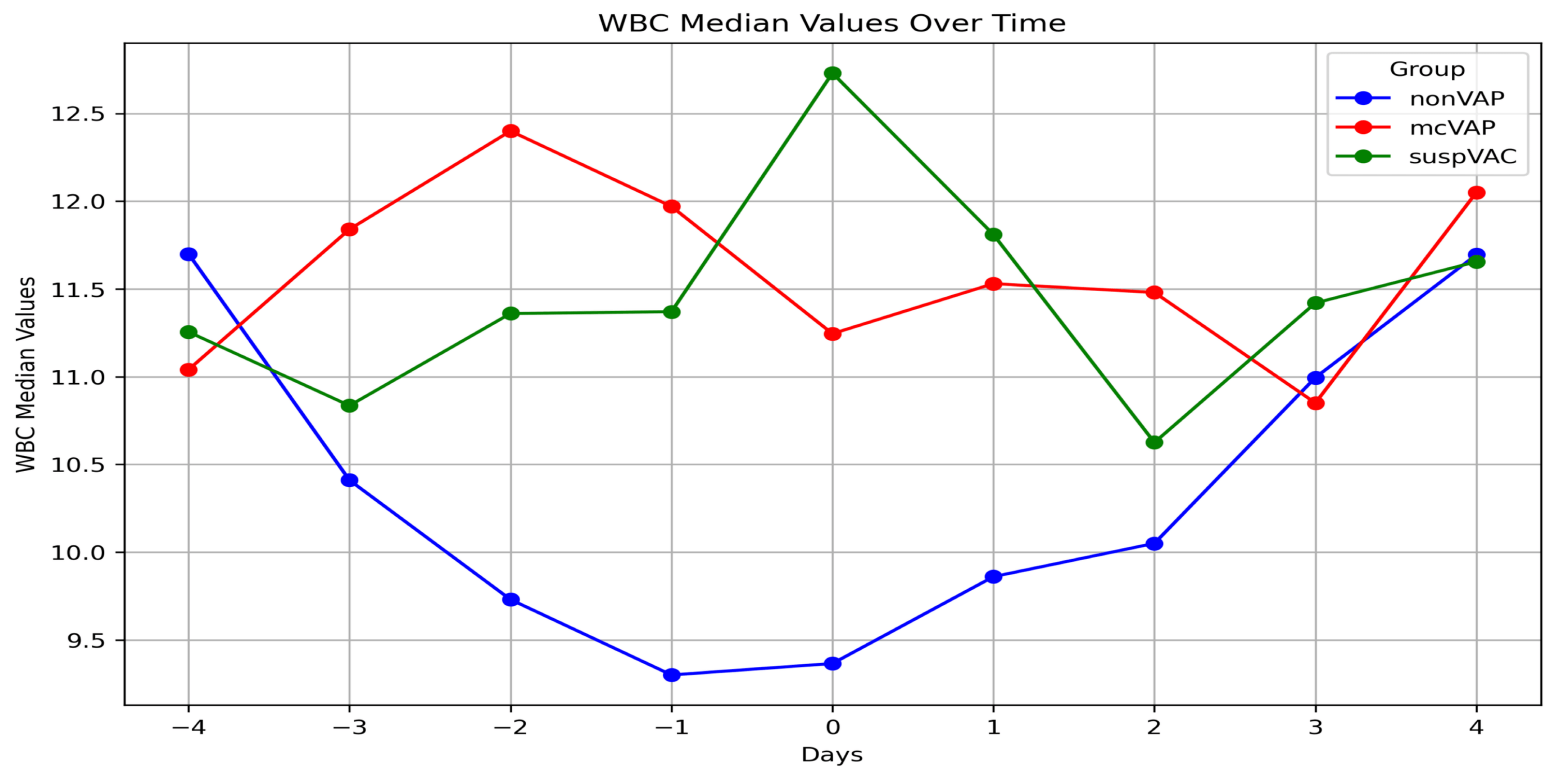
White blood cells in the repetitive assessment The graph illustrates the median WBC levels over time (days -4 to +4) in three patient groups: non-VAP, mcVAP, and suspVAP. Median values are plotted for each day, highlighting trends and differences between groups. WBC: white blood cell, VAP: ventilator-associated pneumonia

**Figure 7:**
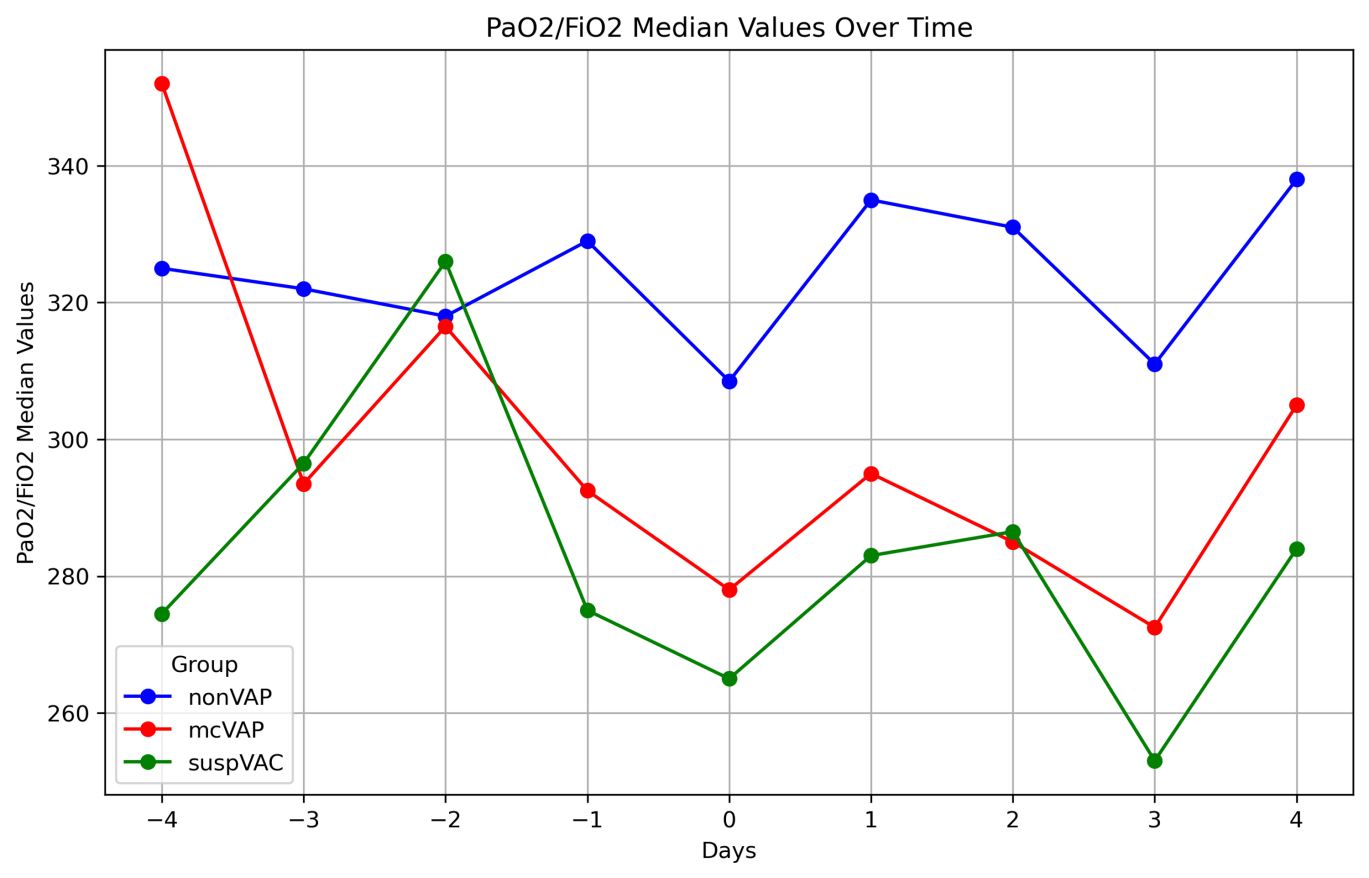
P/F levels in the repetitive assessment The graph illustrates the P/F levels over time (days -4 to +4) in three patient groups: non-VAP, mcVAP, and suspVAP. Median values are plotted for each day, highlighting trends and differences between groups. VAP: ventilator-associated pneumonia

While the IL-6 levels decreased continuously in the non-VAP group, in the suspVAP and mcVAP groups, the IL-6 level increased again from day -1, reached its peak on day 0, and decreased again after the administration of an antibiotic. Compared with those in non-VAP patients, the median levels of IL-6 in mcVAP and suspVAP patients were significantly elevated starting the day before diagnosis (Figure 3). Additionally, we detected significant differences in PCT and CRP levels on days one, two, and three (Figures 3-7, Suppl. Table 4).

Specificity and sensitivity

**Figure 8:**
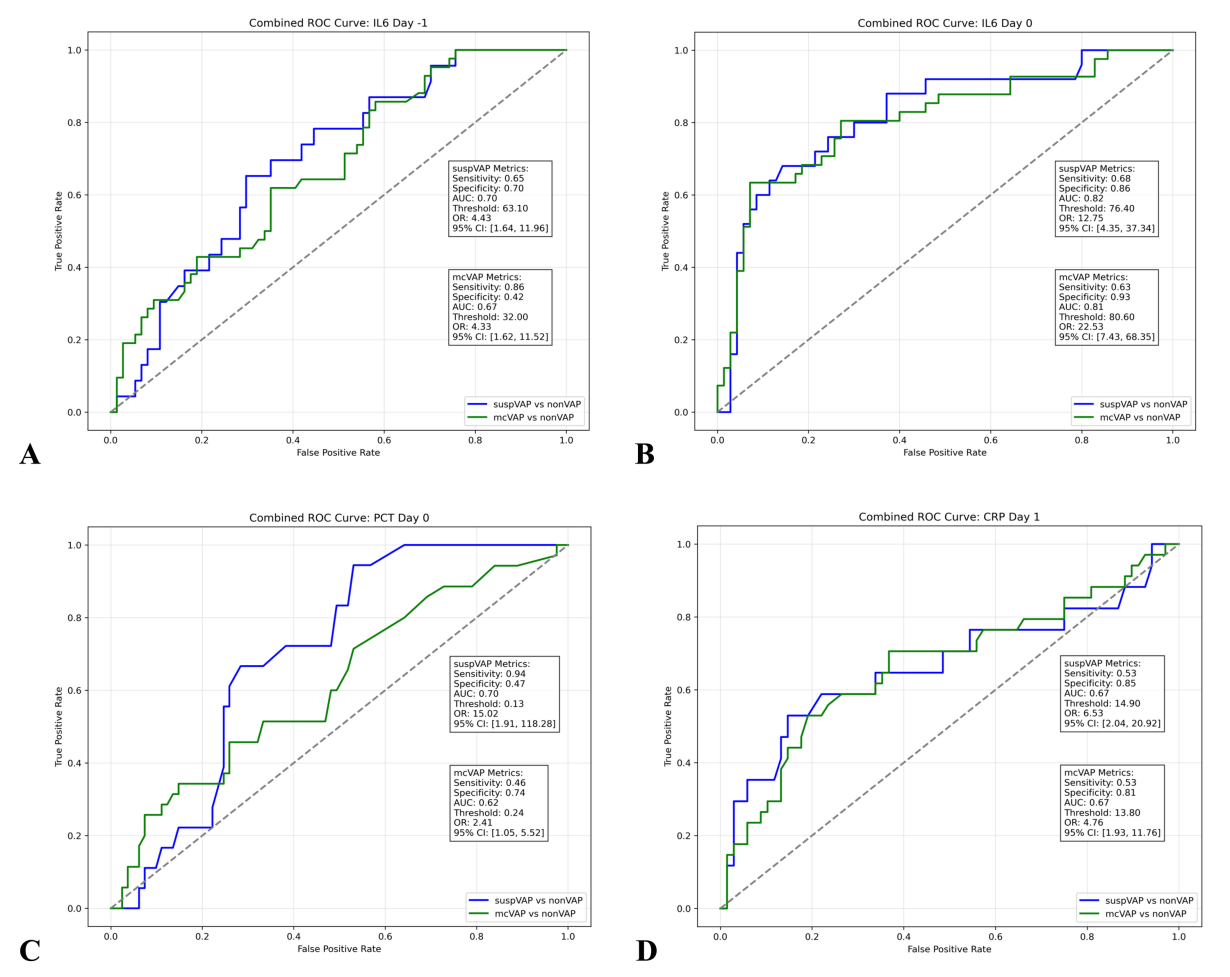
ROC curves for IL-6, PCT, and CRP The combined ROC curve was used to compare the diagnostic performance of A) IL-6 on day -1, B) IL-6 on day 0, C) PCT on day 0, and D) CRP on day 1 based on their significance levels for differentiating between suspVAP and non-VAP patients and between mcVAP and non-VAP patients. The sensitivity, specificity, area under the curve (AUC), and odds ratios (ORs) with 95% confidence intervals are displayed for each comparison. The dashed diagonal line represents the line of no discrimination (AUC=0.5). IL-6: interleukin-6, PCT: procalcitonin, CRP: C-reactive protein, ROC: receiver operating characteristic, WBC: white blood cell, VAP: ventilator-associated pneumonia

ROC analysis revealed that IL-6 discriminated mcVAPs and suspVAPs from non-VAP with AUCs of 0.81 and 0.82, respectively, on day 0 and 0.72 and 0.68, respectively, on day -1. PCT had AUCs of 0.62 and 0.7 on day 0. CRP reached significance on day one, with an AUC of 0.67 for both mcVAP and suspVAP (Figure 8).

Comparison of clinical decision and outcome parameters between the IL-6 and PCT/CRP groups

For further analysis, the cohort was divided into an IL-6 group (n=155) and a PCT/CRP group (n=85). Here, we observed that, in the IL-6 group, the mcVAP and suspVAP were not treated earlier than they were in the PCT/CRP group. The duration of antibiotic administration was slightly shorter in the IL-6 group than in the control group, but the differences were not significant. Ventilation duration also showed no significant differences. However, the patients in the suspVAP group in the PCT/CRP cohort had a significantly longer intensive care unit (ICU) stay (Table 3).

**Table 3:** Comparison of clinical decision and outcome parameters between the IL-6 and PCT/CRP groups Comparison of key clinical parameters between the IL-6 group (n=155) and the PCT/CRP group (n=85) for patients with mcVAP and suspVAP. The values are presented as means (± standard deviations). A significant difference was only observed in the length of stay in the PCT/CRP group among patients with suspected VAP (p=0.003*). IL-6: interleukin-6, PCT: procalcitonin, CRP: C-reactive protein, WBC: white blood cell, VAP: ventilator-associated pneumonia

Cohort	IL-6 Group (n=155)	PCT/CRP Group (n=85)	p-value
mcVAP – Day of Recognition	5.36 (±3.96)	6 (±3.62)	0.373
suspVAP – Day of Recognition	6.59 (±4.98)	6.0 (±2.65)	0.790
mcVAP - Length of AB (days)	6.25 (±3.73)	7.37 (±4.95)	0.339
suspVAP - Length of AB (days)	5.9 (±2.32)	7.67 (±2.08)	0.214
mcVAP - Length of Ventilation (h)	382 (±239.21)	387 (±212.14)	0.940
suspVAP - Length of Ventilation (h)	323 (±177.83)	342 (±75.11)	0.813
mcVAP - Length of ICU Stay (h)	564 (±369.53)	614 (±284.54)	0.594
suspVAP - Length of ICU Stay (h)	498 (±182.8)	883 (±354.34)	0.003*

## Discussion

The first step in identifying VAP is suspicion by the physician in charge. This first observation includes a variety of clinical criteria, examinations, and laboratory diagnostics (fever, secretion load, change in ventilation settings, X-ray diagnostics, bronchoscopy findings, and inflammatory parameters) [23]. Each of these diagnostics alone leaves uncertainty regarding the diagnosis of VAP. In particular, radiological findings, which are mandatory for the definition of VAP, create a large gap between the overdiagnosis and omission of VAP [24-26]. Laboratory markers help heighten our suspicions in the early stages of VAP. To our knowledge, this is the first study to investigate the combination of PCT, CRP, and IL-6 levels and their effect on clinical outcomes.

Despite the shortcomings mentioned above, we used the common VAP definition on the basis of chest X-ray findings combined with at least two of the following criteria: 1) white blood cell count above 10/nL or below 4/nL, 2) fever above 38°C, and/or 3) purulent secretions. We distinguished between microbiologically confirmed VAP (mcVAP) and suspected VAP (suspVAP), which met the clinical criteria but had negative microbiological results.

For surveillance purposes, the CDC introduced the terms infection-related ventilator-associated complication (IVAC) and possible ventilator-associated pneumonia (PVAP) for similar conditions. These definitions require a prior decrease in lung oxygenation capacity. Although we observed a decrease in the P/F ratio in both groups, it was not significant in mcVAPs compared with non-VAPs. As a result, many patients with clinically confirmed and treated VAP would have been reclassified as not having IVAC or PVAP. The second limitation of why we did not use the terms IVAC and PVAP was the retrospective nature of our data. While we had access to the P/F ratio, we lacked information on changes in FiO₂ or PEEP throughout the day, which is required for IVAC and PVAP classification.

In our cohort, VAP occurred mainly on the sixth day after intubation, consistent with previous publications' findings [27]. This makes the period around the sixth day when we should be alert for VAP. Additionally, we have shown that patients with mcVAP and suspVAP were, on average, ventilated five days longer than those without VAP. This is consistent with previous studies' findings, providing evidence for the importance of VAP [1,2,28].

Interestingly, mcVAP and suspVAP behaved very similarly, especially regarding the dynamics of CRP and IL-6. This might be due to their low sensitivity in detecting relevant bacteria, which would explain why the level of IL-6 decreased immediately in both groups after antibiotic administration.

For our clinical practice, we should attach great importance to producing good microbiological material, such as bronchoalveolar lavages (BALs) or protected specimen brushings. Nevertheless, detecting a pathogen should not play a role in starting an antibiotic and may also not be helpful for continuation if the microbiology remains negative. In our study, we routinely obtained samples from deep tracheal secretions. In some cases with a high mucus load, BAL was performed. Since the sensitivity of BAL is higher than that of tracheal specimens, this may have introduced a bias in differentiating between mcVAP and suspVAP.

In the future, using bedside electronic nose sensor array signals and/or nanopore sequencing might help with the early detection of relevant bacteria [29,30].

Among all the markers we tested, IL-6 increased one day before mcVAP and suspVAP, had the greatest effect size on the day of VAP, and decreased rapidly after antibiotics were administered. Although we could not prove that PCT was elevated the day before mcVAP and suspVAP, PCT significantly predicted both on day 0, with a very low threshold below 0.5 mg/L. Therefore, we consider PCT, in addition to IL-6, a reliable surrogate marker when it is increased on the day of VAP. Nevertheless, VAP was more often diagnosed in the IL-6 group, raising the question of whether IL-6 leads to false-positive diagnoses or PCT/CRP leads to false-negative diagnoses. To answer this, further studies are needed. 

Other investigators have identified CRP as a sensitive marker for infection in the ICU setting [25]. In our study, CRP demonstrated significance on days one and two following the onset of mcVAP and suspVAP, aligning with its slower response to stimuli. This delayed increase may contribute to late recognition of VAP; however, in our cohort, we could not detect any delay in comparing CRP and IL-6. Additionally, the continued rise in CRP levels on the second day after initiating antibiotic therapy could lead to premature changes in antibiotic regimens on the basis of the misinterpretation of trends.

We found no significant difference between the IL-6 and PCT/CRP groups regarding the timing of mcVAP and suspVAP diagnoses. These findings suggest that other factors, such as clinical signs, may play a more critical role in diagnosing VAP. However, we observed a slight trend toward shorter durations of antibiotic administration in the IL-6 group, which could be attributed to the rapid decline in IL-6 levels following antibiotic therapy.

The strength of this study is that we selected a very distinct cohort of patients who mainly presented as emergencies and thus were not exposed long-term to hospital germs, hospital-acquired weakness (including dysphagia), chronic inflammation, and/or repetitive operations before inclusion in the study.

A key limitation of our study lies in the retrospective nature of the data collection, which inevitably leads to missing information, particularly laboratory data. Another limitation, as previously discussed, is the unsuitability of the new CDC definition for our study. Despite the lack of data required to apply this definition, it is essential to note that this definition is explicitly not intended as a clinical algorithm. Given that our study was designed from a clinician's perspective, this definition would not have been appropriate for our purposes. In neurosurgical patients, good oxygenation is essential. To diagnose IVAC, an increase in PEEP or FiO₂ is mandatory, which might lead to an underdiagnosis of VAP, as the increase is not necessarily linked to an alteration in lung function. Additionally, for PVAP, microbiological confirmation is required, which would have resulted in the underrecognition of 30 VAP patients. However, for future prospective studies, it may be useful to compare these approaches for the early detection of VAP. Statistically, one limitation might be the variation in tests. However, to do justice to the study objectives, this variety was necessary to appropriately address the distribution of the target values. Another limitation is that the marker that performed best in our study is the least available and most expensive. However, integrating it into a protocol-driven decision-making process may have the potential to reduce antibiotic exposure and complications, ultimately leading to cost savings in the long run. Additionally, targeted testing when a patient is worsening might reduce costs; however, to our understanding, it is less sensitive, as the dynamic changes over time are of great importance. However, it is worth noting that some hospitals already use IL-6 as a single marker for inflammation in the ICU, which may provide more real-world data in the future.

## Conclusions

Our study emphasizes the complexity of the diagnosis of VAP, in which the individual diagnostic methods have their limitations. Radiological criteria, while central to the VAP definition, pose challenges due to their potential for both overdiagnosis and omission. Laboratory markers, particularly IL-6, have demonstrated significant utility in the early identification of patients fulfilling the clinical criteria for VAP. However, its direct impact on clinical outcomes remains inconclusive. Our study also highlights critical limitations, including the retrospective design, incomplete data, and challenges in applying newer definitions such as IVAC and PVAP to our cohort. Additionally, the cost and limited availability of IL-6 testing may restrict its widespread use, despite its demonstrated efficacy.

Future research should focus on integrating biomarkers into a protocol-driven decision-making process and analyzing their potential to reduce antibiotic exposure and ICU stay. Further, novel diagnostic tools, such as electronic nose sensors or nanopore sequencing, should be studied to enhance the early detection of VAP. Combined with reliable biomarkers such as IL-6 and PCT, these technologies could help bridge the diagnostic gap and improve patient outcomes. Meanwhile, the emphasis should remain on ensuring high-quality microbiological sampling and carefully interpreting clinical and laboratory findings to guide timely and appropriate antibiotic treatment.

## Appendices

**Table 4:** Biomarker trends (IL-6, WBC, PCT, CRP) in non-VAP vs. suspected/confirmed VAP groups Median (IQR), mean (SD) values of IL-6, WBC, PCT, and CRP over days -4 to +4. P-values < 0.05 (*) indicate significant differences. Effect sizes (rs) show the strength of the correlation. Negative values indicate higher levels in the VAP group. IL-6: interleukin-6, PCT: procalcitonin, CRP: C-reactive protein, WBC: white blood cell, VAP: ventilator-associated pneumonia

Group	Day	non-VAP		SuspVAP and mcVAP		p-value (effect size)
		Median (IQR)	Mean (SD)	Median (IQR)	Mean (SD)	
							IL-6	-4	62.30 (70.58)	93.06 (114.03)	62.20 (76.35)	92.99 (93.37)	0.541
-3	51.70 (72.95)	87.89 (126.92)	50.60 (56.50)	112.06 (291.39)	0.933		
-2	44.30 (61.00)	83.37 (145.22)	41.50 (59.55)	85.66 (120.41)	0.649		
-1	43.00 (52.93)	114.14 (507.68)	63.10 (88.25)	127.64 (187.73)	<0.001* (r_s_=-0.347)		
0	32.55 (32.33)	58.87 (115.39)	104.50 (168.55)	197.55 (249.88)	<0.001* (r_s_=-0.631)		
1	27.80 (30.55)	56.54 (104.64)	63.80 (104.93)	142.75 (213.6)	<0.001* (r_s_=-0.443)		
2	27.30 (27.80)	53.52 (71.26)	45.80 (40.90)	105.72 (242.84)	0.005* (r_s_=-0.204)		
3	24.40 (44.60)	62.43 (111.13)	41.10 (52.00)	114.03 (243.75)	0.023* (r_s_=-0.247)		
4	23.40 (28.40)	48.70 (93.96)	29.55 (36.35)	74.73 (141.61)	0.029* (r_s_=-0.245)		
WBC	-4	11.70 (6.96)	12.82 (6.60)	11.24 (6.01)	12.09 (4.77)	0.620
	-3	10.41 (5.24)	11.50 (5.84)	11.73 (6.01)	12.01 (5.34)	0.220
	-2	9.72 (5.20)	10.46 (4.50)	12.20 (6.29)	12.29 (4.74)	<0.001* (r_s_=-0.267)
	-1	9.20 (4.59)	10.35 (5.31)	11.57 (5.71)	12.1 (4.18)	<0.001* (r_s_=-0.308)
	0	9.16 (4.56)	10.67 (6.42)	12.01 (5.38)	12.36 (4.2)	<0.001* (r_s_=-0.345)
	1	9.72 (4.40)	11.01 (5.89)	11.72 (6.21)	12.34 (4.3)	0.002* (r_s_=-0.258)
	2	10.05 (4.89)	11.68 (6.41)	11.23 (4.57)	12.18 (4.65)	0.086
	3	11.00 (6.27)	12.78 (6.95)	11.28 (5.66)	12.44 (5.18)	0.881
	4	11.60 (5.48)	13.29 (8.18)	11.79 (5.88)	13.15 (5.6)	0.624
PCT	-4	0.2 (0.58)	1.25 (3.82)	0.20 (0.61)	2.41 (11.46)	0.782
	-3	0.2 (0.36)	1.85 (11.43	0.16 (0.48)	0.43 (0.58)	0.785
	-2	0.18 (0.33)	0.69 (1.92)	0.17 (0.25)	0.68 (2.27)	0.460
	-1	0.16 (0.20)	0.95 (5.89)	0.21 (0.55)	3.09 (19.59)	0.073
	0	0.15 (0.21)	1.17 (7.66)	0.24 (0.35)	4.09 (27.46)	0.008* (r_s_=-0.270)
	1	0.11 (0.12)	0.23 (0.36)	0.23 (0.25)	2.59 (15.19)	<0.001* (r_s_=-0.353)
	2	0.12 (0.17)	1.67 (10.1)	0.27 (41)	2.04 (9.92)	<0.001* (r_s_=-0.403)
	3	0.14 (0.140)	1.61 (10.97)	0.20 (0.33)	1.52 (5.48)	0.004* (r_s_=-0.312)
	4	0.13 (0.27)	1.67 (8.5)	0.21 (0.29)	1.33 (4.57)	0.017* (r_s_=-0.268)
CRP	-4	9.35 (6.73)	10.29 (6.2)	4.60 (8.70)	6.35 (7.24)	<0.001* (r_s_=0.437)
	-3	12.3 (9.70)	12.71 (7.65)	6.50 (12.10)	8.53 (8.95)	<0.001* (r_s_=0.359)
	-2	11.3 (10.80)	13.41 (8.95)	6.30 (12.20)	8.91 (8.85)	<0.001* (r_s_=0.343)
	-1	13 (10.35)	13.19 (9.17)	11.15 (10.68)	12.84 (10.44)	0.570
	0	10.1 (8.83)	11.59 (7.14)	12.30 (9.65)	13.77 (8.72)	0.163
	1	8.5 (7.55)	9.79 (6.17)	13.80 (14.53)	14.39 (8.34)	0.002* (r_s_=-0.330)
	2	8.3 (8.60)	8.66 (6)	15.50 (13.70)	16.49 (9.85)	<0.001* (r_s_=-0.482)
	3	6.3 (8.63)	8.07 6.44)	11.35 (10.78)	12.64 (7.43)	<0.001* (r_s_=-0.382)
	4	6.45 (9.18)	8.76 (7.34	8.90 (9.95)	10.67 (8.69)	0.283
